# Evaluation of the effect of sustained‐release progesterone injection on the expression of interferon‐related genes in repeat‐breeder dairy cows

**DOI:** 10.1002/vms3.70005

**Published:** 2024-08-27

**Authors:** Jahangir Modaresi, Ali Kadivar, Naser Shams Esfandabadi, Pegah Khosravian, Abdonnaser Mohebbi

**Affiliations:** ^1^ Department of Clinical Sciences Faculty of Veterinary Medicine Shahrekord University Shahrekord Iran; ^2^ Research Institute of Animal Embryo Technology Shahrekord University Shahrekord Iran; ^3^ Medicinal Plants Research Center Basic Health Sciences Institute Shahrekord University of Medical Sciences Shahrekord Iran

**Keywords:** ISG15, MX1, MX2, repeat‐breeder cow, sustained‐release progesterone

## Abstract

**Background:**

Repeat‐breeder cows repeatedly fail to conceive after at least three attempts and return to oestrus at apparently normal intervals. Repeat‐breeder cows cause economic losses in dairy farms in different ways.

**Objective:**

In the present study, we investigated the effect of sustained‐release progesterone injection in two different doses on the expression of interferon‐related genes in repeat‐breeder dairy cows.

**Methods:**

A total of 96 repeat‐breeder primiparous and multiparous cows were assigned among three groups: control group, inseminated and do not receive progesterone treatment; P_400_ and P_600_ groups, inseminated and received a single‐intramuscular injection of 400 and 600 mg slow‐release progesterone 5 days after insemination, respectively. Blood sampling was carried out on Day 20 after AI for progesterone measurement and evaluation of gene expression for ISG15, MX1 and MX2 genes.

**Results:**

One injection of sustained‐release progesterone increased the expression of ISG15, MX1 and MX2 genes with differences between two different progesterone concentrations. For all three genes, the level of gene expression was higher in progesterone‐supplemented group than in control group, when P_400_ and P_600_ groups considered together. The level of MX2 gene expression was significantly higher in pregnant cows than non‐pregnant cows. There was a significant positive correlation between expression level of all three genes and blood progesterone concentration. The expression level of ISG15 gene showed a significant positive correlation with MX1 and MX2 gene expression.

**Conclusion:**

The use of this sustained‐release progesterone is simple and can be used in repeat‐breeder cows to improve fertility.

## Graphical abstract

One injection of sustained‐release progesterone increased the expression of ISG15, MX1 and MX2 genes in progesterone‐supplemented groups.

The level of MX2 gene expression was significantly higher in pregnant than non‐pregnant cows.

There was a significant positive correlation between the expression level of all three genes and blood progesterone concentration.

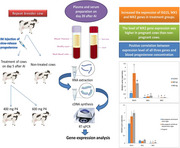

## 1 INTRODUCTION

Progesterone is the main hormone for maintenance and continuity of pregnancy in mammals (Mehni et al., 2012). The level of systemic progesterone influences the uterine secretions, conceptus development rate, production of embryonic interferon‐tau (IFN‐τ) and release of PGF2α (Larson et al., 2007). Poor embryo growth and the inability to inhibit the luteolytic signal are related to low levels of blood progesterone in dairy cattle (Serrano‐Pérez et al., 2019), which leads to early embryonic loss and subsequent reproductive failure (Kasimanickam et al., 2021). Nyman et al. (2018) investigated the relationship of pregnancy loss and milk progesterone concentration in Swedish Red and Swedish Holstein dairy cows and showed that cows with pregnancy loss had significantly lower progesterone concentration at Days 10, 21 and 30 after AI had significantly higher P4 levels at the day of AI compared to pregnant cows. Progesterone supplementation during the post‐conception period has been associated with higher production of IFN‐τ, better conceptus elongation and higher pregnancy rate in cattle (Monteiro et al., 2014).

Presence of early conceptus leads to a process which is named maternal recognition of pregnancy (MRP), in which the embryo signals its presence to the maternal system and prevents luteolysis (Matsuyama et al., 2012). IFN‐τ has been identified as a significant paracrine signal that is secreted by the embryonic trophectoderm at the peri‐implantation stage (Matsuyama et al., 2012). The secretion of IFN‐τ initiates the MRP process in cattle through down‐regulation of oestrogen and oxytocin receptors (Spencer & Bazer, 1996), which leads to reduction in pulsatile secretion of PGF_2α_ (Hansen et al., 2017). In bovine embryo, the secretion of IFN‐τ begins at the morula and blastocyst stages and steadily increases until the trophoblast cells adhere to the endometrium on Day 16. Embryonic IFN‐τ reaches to peak between Days 14 and 17 and declines after implantation (Kowalczyk et al., 2021).

IFN‐τ binds to type I interferon alpha receptor leading to the transcription of various interferon‐stimulated genes (ISGs) such as myxovirus resistance 1 and 2 (MX‐1 and MX‐2) and ISG‐15 (Oli, 2022). Embryonic IFN‐τ diffuses into circulation during the early stages of pregnancy and affects expression of various ISGs in peripheral blood leukocytes (Kaya et al., 2017). In this regard, Gifford et al. (2007) reported that the expression of ISG‐15 on Day 18, MX1 on Day 20 and MX2 from Days 16 to 20 after AI increased in peripheral blood leukocytes isolated from pregnant cows. Other research studies also reported the similar results (Green et al., 2010; Pugliesi et al., 2014).

In repeat‐breeder cow syndrome, cows repeatedly fail to conceive after at least three attempts and return to oestrus at apparently normal intervals (Pérez‐Marín & España, 2007). Repeat‐breeder cows cause economic losses in dairy farms in different ways. Increase in open days and decrease in milk production, decrease in calf production, veterinarian expenses, therapeutic drugs expenses, culling and replacement are part of these economic losses (Pérez‐Marín & Quintela, 2023). It has been found that increase in progesterone concentration in early dioestrus stimulates conceptus development (Carter et al., 2008) and increases IFN‐τ concentration in the uterine lumen (Mann et al., 2006). According to this, progesterone has been used in various studies as one of the solutions to increase fertility in repeat‐breeder cows (Izumi et al., 2020). In our recent study, we developed an injectable slow‐release progesterone formulation and used it for oestrus synchronization in ewes in non‐breeding season (Vafaei Salarpoor et al., 2023). In this formulation, progesterone was made sustained‐release using Eudragit RS100, acetone, Tween 80 and poly‐ethylene glycol 400. In the present study, we investigated the effect of sustained‐release progesterone injection in two different doses on the expression of interferon‐related genes in repeat‐breeder dairy cows and compared it with the untreated group.

## 2 MATERIALS AND METHODS

### 2.1 Preparation of slow‐release progesterone

Slow‐release progesterone was prepared according to the method explained in our the previous study (Vafaei Salarpoor et al., 2023). In this formulation, commercial Eud (Eudragit RS100) was used from Evonik Industries.

### 2.2 Animals, experimental design and blood sampling

This study was conducted on Holstein‐Friesian dairy cows in a commercial dairy farm with around 700 lactating cows (Isfahan Shir livestock and agriculture company). The selection and entry of cows into the study were carried out in November to January 2021. Cows were milked three times daily, fed a total mixed ration and had the standard herd‐health and reproductive management. Ultrasonographic examinations of ovaries and uterus were performed for all postpartum cows at around 30 days after parturition and detected reproductive diseases, for example, endometritis, ovarian cysts and … were treated. Cows were inseminated after voluntary waiting period based on the observed oestrus, and pregnancy diagnosis was performed by ultrasonography between 30 and 35 days after insemination. Non‐pregnant cows were divided into two groups: Cows were non‐pregnant and showed oestrus signs between 18 and 24 days after AI, and cows did not show oestrus signs at this time period.

A total of 96 repeat‐breeder primiparous and multiparous cows were included in the study. Cows that repeatedly fail to conceive after at least three attempts, without any infectious diseases or anatomical abnormalities in reproductive system and return to oestrus at apparently normal intervals were considered repeat breeder (Pérez‐Marín & España, 2007). The mean ± SE values of parity, days in milk, body condition score at the onset of dry period, body condition score after parturition and days open for pregnant cows were 2.96 ± 0.15, 205.43 ± 7.20, 3.5 ± 0.04, 3.16 ± 0.03 and 213.89 ± 8.98. The repeat‐breeder cows were assigned among three groups: (a) control group, including 35 animals, inseminated and did not receive progesterone treatment; (b) P_400_ group, including 31 animals, inseminated and received a single‐intramuscular injection of 400 mg slow‐release progesterone 5 days after insemination and (c) P_600_ group, including 30 animals, inseminated and received a single‐intramuscular injection of 600 mg slow‐release progesterone 5 days after insemination. Pregnancy was diagnosed by ultrasonography between Days 30 and 35 after AI.

To separate buffy coat and to evaluate progesterone concentration, blood sampling was carried out on Day 20 after AI. Blood samples (5 mL) were collected from the tail vein (vena coccygeal) of cows using 18G needle and syringe. To separate buffy coat, blood samples were stored in blood collection tubes containing potassium ethylen diamine tetra acetic acid, placed on ice and transported to laboratory as soon as possible. Samples were centrifuged at 3000 rpm for 15 min, and buffy coats were separated and stored at −70°C until RNA extraction. To separate serum for progesterone evaluation, blood samples were stored in tubes with clot activator, placed on ice and transported to laboratory as soon as possible. Samples were centrifuged at 4000 × *g* for 15 min, and the sera were stored at −70°C until analysis.

### 2.3 Blood progesterone measurement

Serum progesterone concentration was measured by ELISA method using progesterone ELISA Kit 96t (Ideal Tashkhis Atieh) and microplate reader (Roys Anthos 2010, Rosys Labtech). The optical density of each well was read at 450 nm against a 620 nm reference filter. In each progesterone diagnostic kit, progesterone was measured in triplicates for each standard sample (six samples) and each control sample (two samples). Sensitivity was 0.12 ng/mL, and intra‐assay (within run) and inter‐assay (between run) coefficients of variation were 4.3% and 4.9%, respectively, for standard and control samples.

### 2.4 Gene expression analysis

#### 2.4.1 RNA extraction and cDNA synthesis

Total RNA extraction was performed from 150 µL of buffy coat using FavorPrep Blood/Cultured Cell Total RNA (Favorgen) kit according to the user manual. Briefly, buffy coat samples were centrifuged at 300 × *g* for 5 min at 4°C, and the supernatant was removed. Then, 350 µL of FARB buffer and 3.5 µL of ß‐mercaptoethanol were added and vortexed. The sample mixture was filtered through a filter column by centrifugation at full speed for 2 min. The clarified supernatant was transferred to a new microcentrifuge tube, mixed with the same volume of 70% ethanol and filtered through a filter column by centrifugation at full speed for 1 min. The flow‐through was discarded, and the filter was washed three times with two different washing buffers and dried by centrifugation at full speed for 3 min. The extracted RNA was eluted in 40 µL RNase‐free distilled water and immediately used for cDNA synthesis.

The amount and quality of RNA were determined by spectrophotometry (LSPR spectrophotometer MPANM96, Nanomabna), and RNA with an absorbance ratio (A_260/280_) greater than 1.8 was used for cDNA synthesis. The addScript cDNA Synthesis Kit (Addbio) was used to cDNA synthesis in 10 µL total volume: 5 µL of 2× reaction buffer, 1 µL of 10 mM dNTP mixture, 0.5 µL of 10× oligo dT_20_, 0.5 µL of 10× random hexamer and 3 µL of total RNA (around 50 ng total concentration). The temperature cycling protocol was priming at 25°C for 10 min, then incubation at 50°C for 60 min for reverse transcription (RT) and incubation at 80°C for 5 min for RT inactivation and finally holding at 12°C. The cDNA was stored at −30°C until performing quantitative real‐time PCR.

#### 2.4.2 Quantitative real‐time PCR

Quantitative real‐time PCR was performed for each sample using Rotor‐Gene Q 6000 (Qiagen). The information of primers is shown in Table 1 (Kizaki et al., 2013; Yoshino et al., 2018). The reaction was performed using RelQ Plus 2× Master Mix Green (Ampliqon) according to the manufacturer's recommendations. The reaction was prepared in 10 µL total volume: 5 µL of RealQ Plus 2× Master Mix, 0.5 µL (0.2 µM) of forward and reverse primers and 0.5 µL of cDNA and 3.5 µL of PCR‐grade H_2_O. The reaction condition was holding at 95°C for 15 min, and 40 cycles of thermocycling, including 95°C for 15 s, 60°C for 30 s and 72°C for 30 s for all four primers. In the end, melting curve analysis was performed to ensure the product homogeneity by continuously recording the fluorescence signals during the temperature ramp (70–95°C). The no‐template control was used to check contamination in the PCR reagents and during reactions preparation.

**TABLE 1:** Characteristics of used primers.

Gene name	Primer sequence 5′–3′	Accession no.	Amplicon size (bp)
ISG15	Forward: GCAGACCAGTTCTGGCTGTCT Reverse: CCAGCGGGTGCTCATCAT	NM_174366	58
MX1	Forward: GAGGTGGACCCCCAAGGA Reverse: CCACCAGATCGGGCTTTGT	NM_173940	58
MX2	Forward: GGGCAGCGGAATCATCAC Reverse: CTCCCGCTTTGTCAGTTTCAG	NM_173941	55
GAPDH	Forward: AAGGCCATCACCATCTTCCA Reverse: CCACCACATACTCAGCACCAGCAT	NM_001034034.2	76

Abbreviation: GAPDH, glyceraldehyde‐3‐phosphate dehydrogenase; ISG15, interferon‐stimulated gene 15.

To normalize, the differences between samples, including the input load of cDNA, glyceraldehyde‐3‐phosphate dehydrogenase (GAPDH), was selected as the housekeeping gene. To determine the mean efficiency values (*E*) for each gene, the amplification profiles of individual samples were analysed by LinRegPCR software version 2012.0 (Ruijter et al., 2009). The mRNA level of each target gene relative to GAPDH was estimated for each sample in control, P_400_ and P_600_ groups by the following formula: $\frac{{E_{GAPDH}}^{\left(Ct\;sample\right)}}{{E_{target}}^{\left(Ct\;sample\right)}}.$

Relative gene expression was calculated as following (Dorak, 2006; Pfaffl, 2004): $\frac{{E_{GAPDH}}^{\left(Ct\;sample\right)}}{{E_{target}}^{\left(Ct\;sample\right)}}/\frac{{E_{GAPDH}}^{\left(Ct\;control\right)}}{{E_{target}}^{\left(Ct\;control\right)}}$

Where *E* is mean efficiency value, and *Ct* is the crossing point above background fluorescence.

### 2.5 Statistical analysis

The mRNA level of each target gene relative to GAPDH was calculated for each sample. Mean differences of gene expression among experimental groups were analysed through independent samples *t* test or one‐way analysis of variance (ANOVA). One‐way ANOVA was followed by Tukey's post hoc test. The correlation between expression level of all three genes and blood progesterone concentration and expression level of all three genes with each other was evaluated by Pearson correlation test. The Shapiro–Wilk test was used to evaluate the normality of obtained data. Statistical analysis was performed with SPSS software (version 16; SPSS Inc.). The results are shown as mean ± SEM, and differences were considered significant at *p* < 0.05.

## 3 RESULTS

**FIGURE 1:**
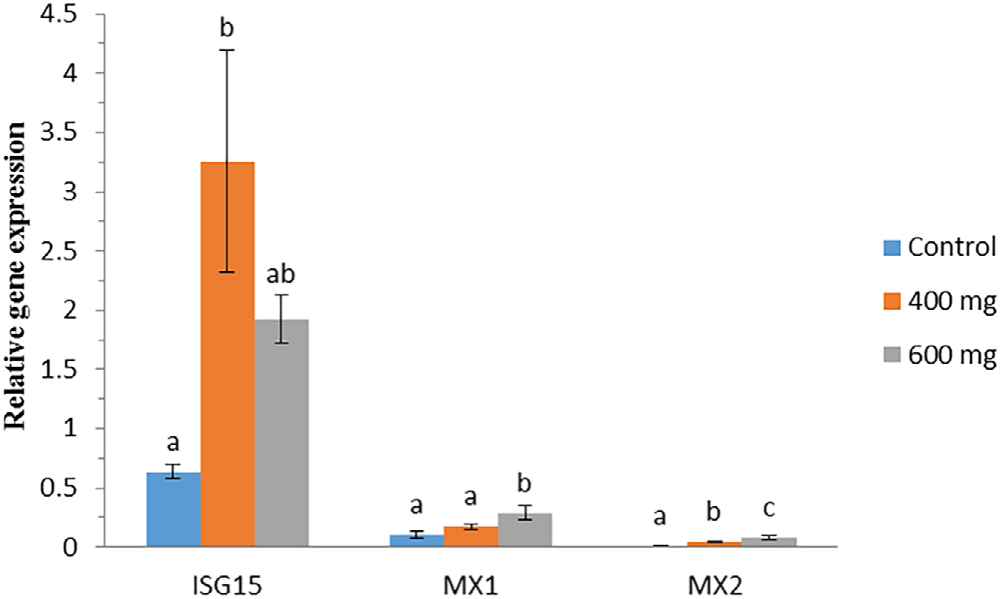
Relative expression of interferon‐stimulated gene 15 (ISG15), MX1 and MX2 genes in control (*n* = 33), P_400_ (*n* = 31) and P_600_ (*n* = 30) groups. Different letters show significant differences between groups in each gene.

Relative expression of ISG15, MX1 and MX2 genes was calculated and compared among control, P_400_ and P_600_ groups. As it is shown in Figure 1, the expression level showed significant differences for all three genes in experimental groups. The expression level of ISG15 gene was significantly higher in P_400_ than control group (*p* < 0.05), but the difference between P_400_ and P_600_ groups and P_600_ and control groups was not significant (*p* > 0.05). For MX1 gene, the expression level was significantly higher in P_600_ group than P_400_ and control groups (*p* < 0.05) and the difference between control and P_400_ groups was not significant (*p* > 0.05). For MX2 gene, the expression level was significantly different among all experimental groups in a way that the expression level was higher in P_600_ group than P_400_ group, which was higher in P_400_ group than control group (*p* < 0.05).

**FIGURE 2:**
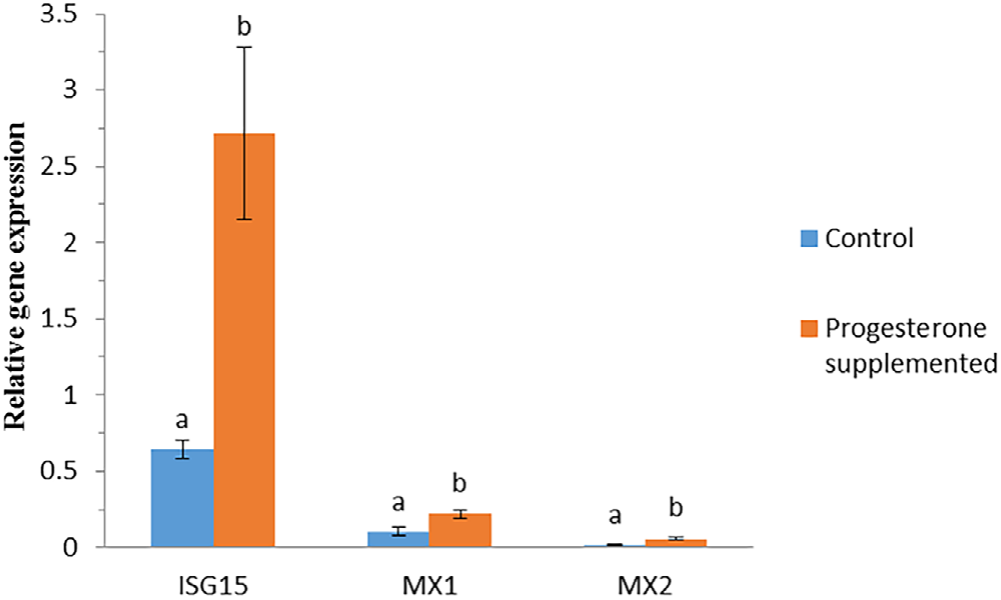
Relative expression of interferon‐stimulated gene 15 (ISG15), MX1 and MX2 genes in progesterone‐supplemented (*n* = 61) and control (*n* = 33) groups. Both P_400_ and P_600_ groups were considered together and compared to control group. Different letters show significant differences between groups in each gene.

The level of relative gene expression was also compared among progesterone‐supplemented and control groups, when P_400_ and P_600_ groups were considered together and compared to control group. The results are shown in Figure 2. For all three genes, the level of gene expression was higher in progesterone‐supplemented group than control group and the differences were significant (*p* < 0.05).

**FIGURE 3:**
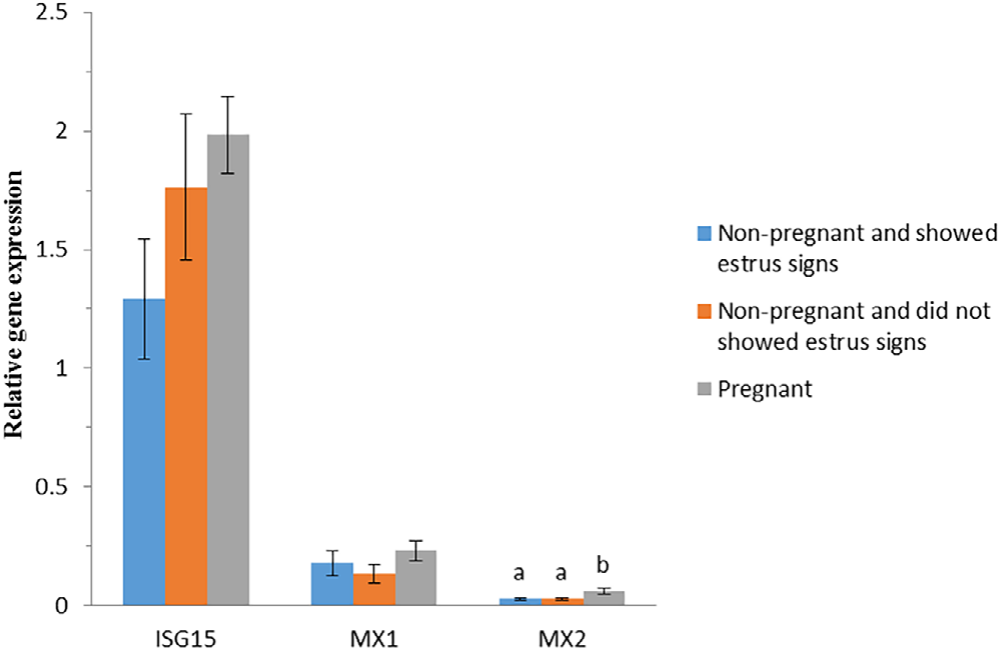
Relative expression of interferon‐stimulated gene 15 (ISG15), MX1 and MX2 genes in pregnant (*n* = 51) and non‐pregnant cows (*n* = 43). Non‐pregnant cows were divided into two groups: Cows were non‐pregnant and showed oestrus signs between 18 and 24 days after AI (*n* = 21), and cows did not show oestrus signs at this time period (*n* = 22). Different letters show significant differences between groups in each gene.

Gene expression level was compared among cows were detected pregnant (*n* = 51) and non‐pregnant (*n* = 43) by ultrasonography between Days 30 and 35 after AI. Two cows were culled before the first pregnancy test. The differences were significant for MX2 gene (*p* < 0.05). The level of MX2 gene expression was significantly higher in pregnant cows than in both two groups of non‐pregnant cows (cows showed oestrus signs [*n* = 21], and cows did not showed oestrus signs [*n* = 22]), but the difference between two groups of non‐pregnant cows was not significant (*p* > 0.05). The level of gene expression for ISG15 and MX1 genes was not significantly different between pregnant and two groups of non‐pregnant cows (*p* > 0.05) (Figure 3).

The correlation between expression level of all three genes and blood progesterone concentration on Day 20 after AI was evaluated. There was a significant positive correlation between expression level of all three genes and blood progesterone concentration (*p* < 0.05). The expression level of ISG15 gene was significantly correlated with MX1 and MX2 genes expression, and the correlation was positive (*p* < 0.05). The correlation between expression level of MX1 and MX2 genes was also significant and positive (*p* < 0.05) (Table 2).

**TABLE 2:** Correlation between expression level of interferon‐stimulated gene 15 (ISG15), MX1, MX2 genes and blood progesterone concentration.

	N		ISG15 GE	MX1 GE	MX2 GE	Progesterone concentration
**ISG15 GE**	94	Pearson correlation	1	0.360	0.440	0.282
		*p*‐Value		0.009	0.002	0.037
**MX1 GE**	94	Pearson correlation		1	0.798	0.283
		*p*‐Value			0.000	0.044
**MX2 GE**	94	Pearson correlation			1	0.287
		*p*‐Value				0.048
**Progesterone concentration**	94	Pearson correlation				1
		*p*‐Value				

*Note*: Progesterone concentration: blood progesterone concentration on day 20 after AI.

Abbreviation: GE, gene expression.

**FIGURE 4:**
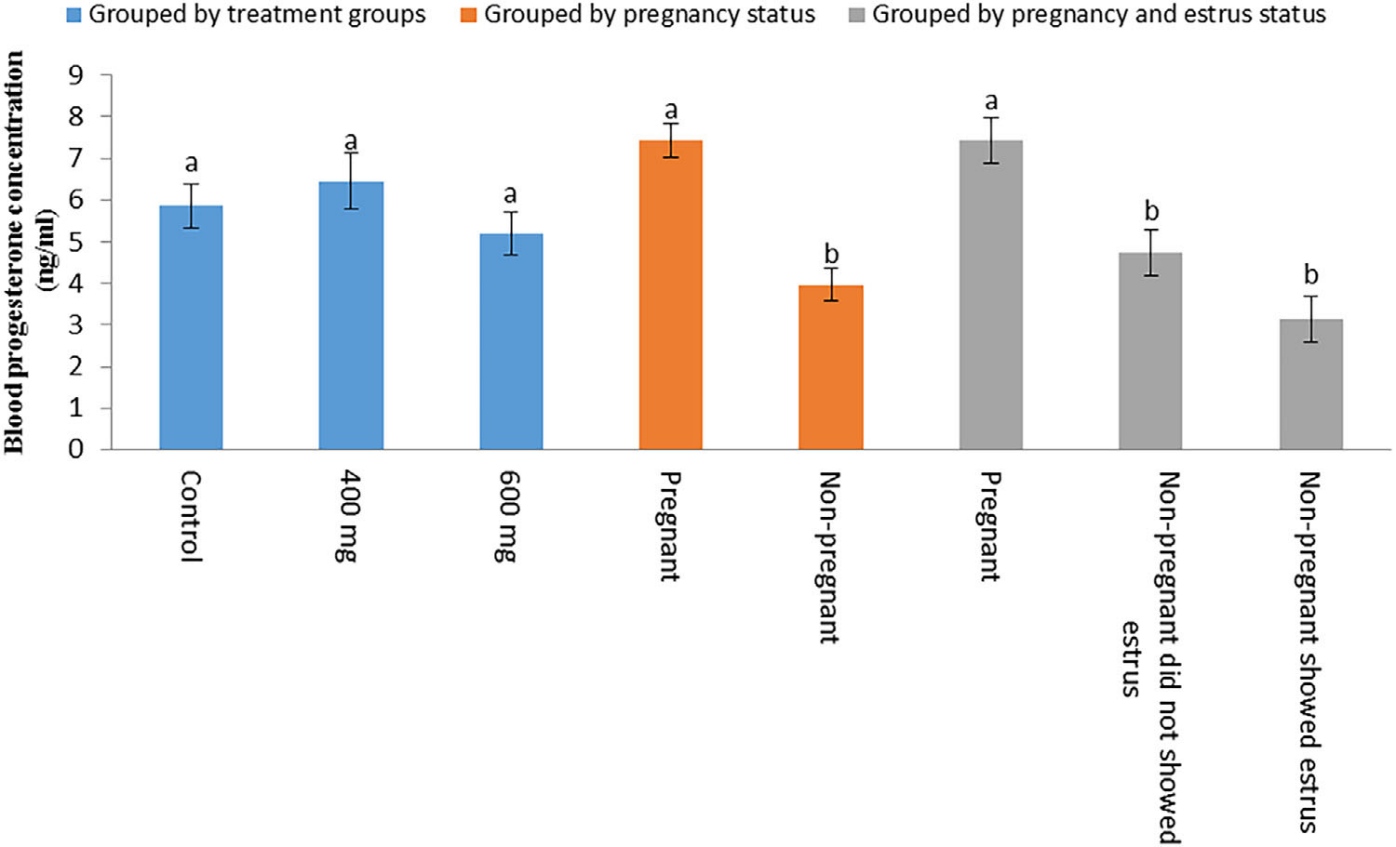
Comparison of blood progesterone concentration in the studied groups: grouped by treatment groups (control [*n* = 33], P_400_ [*n* = 31] and P_600_ [*n* = 30]), grouped by pregnancy status (pregnant [*n* = 51] and non‐pregnant cows [*n* = 43]) and grouped by pregnancy and oestrus status (pregnant [*n* = 51], non‐pregnant did not show oestrus [*n* = 22] and non‐pregnant showed oestrus [*n* = 21]). Different letters show significant differences between groups in each category.

Blood progesterone concentrations (mean ± SE) on Day 20 after AI were 5.86 ± 0.54, 6.45 ± 0.67 and 5.19 ± 0.52 ng/mL in control, P_400_ and P_600_ groups, respectively (Figure 4). The difference of progesterone concentration among treatment groups was not significant (*p* > 0.05). Blood progesterone concentrations (mean ± SE) on Day 20 after AI were also compared among cows were detected pregnant (*n* = 51) and non‐pregnant (*n* = 43) by ultrasonography between Days 30 and 35 after AI, as well as among pregnant cows, cows were non‐pregnant and showed oestrus signs between 18 and 24 days after AI (*n* = 21) and non‐pregnant cows did not show oestrus signs at this time period (*n* = 22) (Figure 4). Blood progesterone concentrations were 7.44 ± 0.33 and 3.97 ± 0.48 ng/mL in pregnant and non‐pregnant cows, respectively, and the difference was significant (*p* < 0.05). Blood progesterone concentrations were 7.44 ± 0.33, 4.75 ± 0.73 and 3.15 ± 0.60 ng/mL in pregnant cows, cows were non‐pregnant did not showed oestrus signs and non‐pregnant cows showed oestrus signs, respectively. The difference among pregnant cows and both groups of non‐pregnant cows (showed oestrus signs and did not show oestrus signs) was significant (*p* < 0.05), but the difference between two groups of non‐pregnant cows was not significant (*p* > 0.05).

The percentage of pregnant cows based on the ultrasonographic examination performed on Days 30–35 after AI was 46.88%, 64.52% and 50.00% in control, P_400_ and P_600_ groups, respectively. Percentage of pregnant cows was not significantly different among treatment groups (*p* > 0.05).

## 4 DISCUSSION

In the present study, we investigated the effect of sustained‐release progesterone injection in two different doses on the expression of interferon‐related genes in repeat‐breeder dairy cows and compared it with the untreated group. In our study, one injection of sustained‐release progesterone 5 days after AI increased the expression of ISG15, MX1 and MX2 genes with differences between two different progesterone concentrations. Blood progesterone concentrations on Day 20 after AI were compared among treatment groups as well as among pregnant and non‐pregnant cows. Although the progesterone concentrations were not significantly different among experimental groups, they were higher in pregnant cows than in both groups of non‐pregnant cows (showed oestrus signs and did not show oestrus signs). When the cows were grouped according to pregnancy, the pattern of difference in progesterone concentration between the groups was consistent with the pattern of ISG expression on this day. In the investigations conducted by the researchers of the current study on the concentrations of 400–600 mg of this sustained‐release progesterone, the length of time of retention of this progesterone in amounts of more than 1 ng/mL in the blood was 8–10 days (unpublished data). Considering that sustained‐release progesterone injection was done on Day 5 after insemination, progesterone concentrations measured in the present study on Day 20 after AI were related to the endogenous progesterone of cows. Therefore, the effect of sustained‐release progesterone does not remain until Day 20 after AI (15 days after injection), and the absence of a significant difference in progesterone concentration among treatment groups on this day is logical.

The percentage of pregnant cows in P_400_ and P_600_ groups was higher than the control group. In addition, this percentage was higher in the P_400_ group than the P_600_ group. The percentage of pregnant cows in the P_400_ group was 14.52% and 18% higher than the P_600_ and control groups, respectively. Although this difference was not statistically significant in the present study, this increase in pregnancy rate has great economic importance in the dairy industry. The results of various studies have shown that progesterone supplementation in early dioestrus on Days 3–7 after AI has increased pregnancy rate. In a review article by Wiltban et al. (2014), the effects of circulating progesterone on dairy cattle reproduction have reviewed. Of the 30 trials that they evaluated, most of them (25/30) like our study showed a numeric improvement in fertility with progesterone supplementation. This improvement was statistically significant only in six of these trials (*p* < 0.05). In our study, pregnancy rate was higher in P_400_ than P_600_ groups. In some studies, the use of progesterone supplements at early dioestrus has shortened the luteal lifespan. The causes of reduced luteal lifespan caused by progesterone supplementation can be negative feedback for LH secretion. At the most stages of dioestrus, LH stimulates function of the corpus luteum (CL). High progesterone concentrations cause a negative feedback in LH secretion (Kojima et al., 2003) and as a result inadequate luteotrophic support, premature CL demise and decrease pregnancy rate occur (Ginther, 1970; O'hara et al., 2016).

Progesterone supplementation after AI is used in cases that is the possibility of a defect in progesterone secretion with the aim of providing sufficient amounts of this hormone and to lengthen exposure of the conceptus and endometrium to sufficient progesterone concentration. Progesterone supplementation after AI with the aim of increasing the pregnancy rate was stated with the study of Mann and Lamming (1999). In their study, progesterone supplementation that was initiated during late metoestrus or early dioestrus but not after Day 6 post‐AI could improve pregnancy per AI. The results of some other studies have also showed that improvement in pregnancy per AI is linked with increase in progesterone concentration in early dioestrus (Parr et al., 2012; Stronge et al., 2005). Based on the results of some studies, increase in progesterone concentration in early dioestrus stimulates conceptus development (Carter et al., 2008; Mann et al., 2006) and increases IFN‐τ concentration in the uterine lumen (Mann et al., 2006). Interferon‐τ produces by the conceptus trophoblast cells. It has local effects on the endometrium as well as exits the uterus, enters the uterine vein (Oliveira et al., 2008) and stimulates ISG expression in leukocytes (Matsuyama et al., 2012).

Considering the economic losses caused by the presence of repeat‐breeder cows, we designed the present study on this group of cows. Yaginuma et al. (2019) investigated the effect of embryo transfer following artificial insemination on pregnancy rate of repeat‐breeder dairy cattle. They showed that this method could improve pregnancy rate of repeat‐breeder cows. In this study, the mRNA expression of ISG15 and MX2 genes in white‐blood cells on Day 14 after oestrus was significantly higher in the AI + embryo transfered group than in the AI + sham group (only embryonic cryopreservation solution was transfered). The mRNA expression of MX1 was not significantly different between these groups. The mRNA expressions of ISG15 and MX2 on Day 21 after oestrus were also significantly higher in the white‐blood cells of pregnant repeat breeders than non‐pregnant repeat breeders. These researchers concluded that adding embryos improves conception rate by supplementing IFN‐τ; therefore, it can be assumed that the production of IFN‐τ from original embryos in repeat breeders is low.

In our study, progesterone supplementation increased the expression of ISG15, MX1 and MX2 genes, 20 days after AI, and the level of MX2 gene expression was significantly higher in pregnant cows than in both two groups of non‐pregnant cows (cows showed oestrus signs, and cows did not showed oestrus signs). The higher progesterone level during the post ovulatory period in pregnant cows induced necessary progesterone‐mediated stimulus to embryo, which leads to the production of sufficient amounts of trophoblast IFN‐τ that are necessary to inhibit luteolysis. Maintenance of progesterone production by the CL increases the chance of normal embryo development, successful embryo implantation and maintaining pregnancy (Lau et al., 1992; Vallet & Lamming, 1991). In addition to inhibiting luteolysis, embryonic IFN‐τ stimulates expression of ISGs both in intra‐uterine and extra‐uterine tissues (Oliveira et al., 2008). Immune cells, including the polymorphonuclear granulocytes, are among the extra‐uterine tissues that are more responsive to IFN‐τ and express ISGs (Shirasuna et al., 2012). There are various studies that show that ISG expression is higher in pregnant cows than the conception failure cows. In the study of Sheikh et al., the expression of ISGs, including ISG15, OAS1, MX1 and MX2, in peripheral blood neutrophils was significantly higher during Days 8–42 post AI compared to day of AI. Days 18–21 showed the highest level of expression in pregnant cows. During Days 4–42 post AI compared to day of AI, ISG expression was also significantly higher in late embryonic mortality cows (Sheikh et al., 2018). Haq et al. reported that IFN‐τ secreted from the trophectoderm increased ISG15 expressionin in peripheral blood mononuclear cells. Moreover, by stimulating the peripheral blood mononuclear cells with IFN‐τ, a dose dependent increase in ISG15 expression and its protein was observed (Haq et al., 2016). Up‐regulation of ISGs in extra‐uterine tissues and peripheral blood mononuclear cells under the influence of IFN‐τ indicated that IFN‐τ goes out of the uterus into the circulation to exert its effects that modify maternal physiology (Bott et al., 2010). Ott et al. suggested that MX protein has GTPase activity. Therefore, in early pregnancy, MX protein might be involved in the calcium metabolism of endometrial (Ott et al., 1999). In the study of Mohammed et al. (2022), the expression of ISGs, including MX1, OAS1 and ISG15 in blood neutrophils significantly, increased during the peri‐implantation period in pregnant cows. As already mentioned, the major function of IFN‐τ is to suppress luteolysis by silencing the expression of oestrogen and oxytocin receptors. Besides, IFN‐τ‐induced up‐regulation of ISGs in blood neutrophils results in a series of cellular events that modulate the functions of neutrophils. Fiorenza et al. investigated the in vitro direct impact of Day 7 bovine embryo on the immune responses of PMNs. In this study, PMNs were directly stimulated by embryo culture media, or IFN‐τ and mRNA expression was evaluated by real‐time PCR. Both embryo culture media and IFN‐τ directly stimulated expressions of ISGs, including OAS1, ISG15 and MX1. These researchers showed that bovine neutrophils can not only amplify IFN‐τ signals but also can transfer these signals to a new population of neutrophils. This event is critical for embryo tolerance and pregnancy establishment in cattle (Fiorenza et al., 2021).

Therefore, progesterone supplementation presumably improves embryos development, which leads to production of more IFN‐τ from embryos. The yield and quality of oocytes and embryos of repeat‐breeder dairy cows are investigated in some studies. For example, in the study of Sood et al. (2017), ovum pickup and IVF were performed for repeat‐breeder and healthy cows. In this study, blastocyst production was markedly lower in repeat‐breeder cows than in healthy cows (Sood et al., 2017). In another study, when in vitro oocyte maturation media was supplemented with follicular fluid of repeat‐breeder Holstein heifers, in vitro nuclear maturation, fertilization and blastocyst yield were significantly reduced (Kafi et al., 2017). Intensity of oestrus behaviour, endocrine patterns of steroid hormones and LH and ovulation timing have also compared between repeat‐breeder and normal cows (Sood et al., 2015). Oestrus duration and the average preovulatory follicle size were similar between two groups, but oestrus was more intense in the repeat‐breeder cows. In this study, repeat‐breeder cows showed increased steroidogenic activity, subdued LH secretory pattern before the LH peak and short prooestrus period. This endocrine pattern can impair oocyte competence and development. In addition to these hormonal disorders around the time of ovulation, which affect oocyte competence, reduce the chances of fertilization or embryo vitality, subfunctional CL and as a result low progesterone concentration during the first week after ovulation are the other factors that cause disturbances in the embryo development and implantation. This leads to early embryo death in the repeat‐breeder cows. Therefore, some other studies, like our study, have investigated the effects of progesterone supplementation after AI in the repeat‐breeder cows. Garcia‐Ispierto and López‐Gatius (2017) examined the effect of progesterone supplementation after AI on the reproductive performance of high‐producing dairy cows. Cows were assigned to control group (no treatment) and two treatment groups: progesterone treatment started 15 days after AI (P4‐D15) and progesterone treatment started 3 days after AI (P4‐D3). Progesterone supplementation was performed with progesterone‐releasing intra‐vaginal device (PRID), containing 1.55 g of progesterone for 3 days. Based on odds ratios, cows in P4‐D3 and cows in P4‐D15 were 1.71 times and 1.4‐fold more likely to conceive than control cows, respectively. In another study, the effect of progesterone supplementation from Days 15 to 17 post‐AI was investigated on the reproductive performance of high‐producing dairy cows. Cows in their 15th day post‐AI were assigned to control (no‐treatment) and treatment groups (PRID DELTA, containing 1.55 g of progesterone for 3 days). Based on odds ratios, the interaction of treatment × previous retained placenta had a significant effect on conception rate. Progesterone‐supplementated cows that had not the history of retained placenta were 1.6 times more likely to be pregnant on Days 28–34 days post‐AI than the remaining cows (Garcia‐Ispierto et al., 2016). Pirokad et al. (2022) investigated the influence of inducing secondary CL on progesterone concentration and fertility in repeat‐breeder dairy cows. In this study, 5 days after fixed‐time artificial insemination, repeat‐breeder cows were divided into two groups: treatment with GnRH (GnRH5‐treated group) or without treatment (GnRH5‐untreated group). Pregnancy rates were significantly greater in GnRH5‐treated group than in the GnRH5‐untreated group, and cows bearing two CLs on their ovaries had a significant greater likelihood of pregnancy than cows bearing only one CL. In the study of Monteiro et al. (2014), the effects of supplemental progesterone after AI on fertility and expression of ISGs (ISG15 and receptor transporter protein‐4 [RTP4] genes) in blood leukocytes were evaluated in lactating dairy cows. Cows allocated to three groups: control, CIDR from Days 4 to 18 after AI (CIDR4), CIDR on Day 4 and another on Day 7 after AI, and both were removed on Day 18 (CIDR4+7). Pregnancy increased the expression of ISG on day 19 of gestation, but progesterone supplementation did not increase mRNA expression for ISG15 and RTP4 on Day 16 after AI and mRNA expression decreased under the effect of progesterone supplementation on Day 19 after AI. In this study, there was no overall effect of treatment on pregnancy per AI on Day 62 after AI, but an interaction between method of AI and level of progesterone was observed for pregnancy per AI. In progesterone‐supplemented groups, cows inseminated following timed AI had higher pregnancy per AI on Day 62 in lower supplementation group (CIDR4) and in cows inseminated following oestrus detection, the use of a second CIDR on Day 7 (CIDR4+7) resulted in greater pregnancy per AI. In this regard, it should be mentioned that there is a basal ISG expression in leukocytes and the expression is relatively greater or lesser individually in different cows (Green et al., 2010). Green et al. used ISG expression in leukocytes for pregnancy diagnosis in dairy cattle within 18–20 days after AI. In one of their experiments, primiparous cows, multiparous cows and nulliparous heifers were tested for ISG expression in the oestrus cycle before AI and then the expression was tested again 18 days after AI. The amount of ISG expression was similar in pregnant and non‐pregnant cattle before AI but increased in pregnant cows. Furthermore, early embryonic death may occur in some of the cows before the first ultrasonography within 30–35 days after AI. Some of the cows diagnosed as non‐pregnant at this time were pregnant cows that had premature foetal death and, therefore, had increased expression of ISGs expression. Wiltbank et al. (2016) showed that even the presence of a nonviable embryo can secrete IFN‐τ, which leads to extension in lifespan of the CL.

As the results of our study and some other studies show, progesterone supplementation during early dioestrus increases expression of ISGs in blood leukocytes. Progesterone supplementation probably improves the quality and viability of the embryo and increases IFN‐τ production by the conceptus trophoblast cells. This is especially important in repeat‐breeder cows. Because the disturbance in early embryonic development is one of the causes of repeat‐breeder syndrome in cow and progesterone supplementation during early dioestrus can improve pregnancy per AI in these cows. The first ultrasonographic examination for pregnancy diagnosis was performed between Days 30 and 35 after AI in the dairy farm where this study was conducted. Therefore, there was no way to detect early embryonic death before this time. Most likely, the pregnancy of some cows that were diagnosed as non‐pregnant between Days 30 and 35 was lost due to early embryonic death. If it was possible to detect early embryonic death before the first pregnancy test, the possible beneficial effect of exogenous progesterone supplementation after AI on pregnancy rate and ISG expression could be better assessed.

## 5 CONCLUSION

In the present study, we investigated the effect of sustained‐release progesterone injection on the expression of ISGs in repeat‐breeder dairy cows. Sustained‐release progesterone was injected in two different doses, and the expression of ISG was compared between treatment and untreated groups. Upon our results, one injection of sustained‐release progesterone 5 days after AI increased the expression of ISG15, MX1 and MX2 genes, which was different between two progesterone concentrations. The level of MX2 transcripts was significantly higher in pregnant compared to non‐pregnant cows. There was a significant positive correlation between expression level of all three genes and blood progesterone concentration. Moreover, significant positive correlations were detected among ISG15, MX1 and MX2 transcriptions. In conclusion, the application of this sustained‐release progesterone significantly increased the ISG expression in white‐blood cells of repeat‐breeder cows 20 days after AI. Because there is a correlation between the expression level of ISGs and the embryo viability, we suggest a single injection of this progesterone supplement in repeat‐breeder cows to improve their fertility.

## AUTHOR CONTRIBUTIONS

Conceptualization and design were performed by Ali Kadivar. Methodology was performed by Jahangir Modaresi, Pegah Khosravian and Abdonnaser Mohebbi. Formal analysis and investigation were performed by Ali Kadivar and Naser Shams Esfandabadi. Writing of original draft was prepared by Jahangir Modaresi. Writing of final draft, review and editing was performed by Ali Kadivar. All the authors read and agreed with the content of the manuscript.

## CONFLICT OF INTEREST STATEMENT

The authors declare no conflicts of interest.

## FUNDING INFORMATION

Applied Research Centre, Vice Chancellor for Research of Shahrekord University (Grant number: 0GRD34M1576).

### ETHICS STATEMENT

This study was approved by the Ethical Committee of Shahrekord University, Shahrekord, Iran (IR.SKU.REC.1400.002).

### PEER REVIEW

The peer review history for this article is available at https://publons.com/publon/10.1002/vms3.70005.

## Data Availability

The data that support the findings of this study are available from the corresponding author upon reasonable request.
